# Pomalidomide re-exposure after drug-related severe hypothyroidism: a case report

**DOI:** 10.1007/s00277-026-06904-6

**Published:** 2026-02-20

**Authors:** Francesca Bartolomei, Tommaso Za, Francesca Di Landro, Valerio De Stefano, Elena Rossi

**Affiliations:** 1 Section of Hematology, Department of Radiological and Hematological Sciences, Catholic University, Fondazione Policlinico Gemelli IRCCS, Rome, Italy; 2 UOC Servizio e Day Hospital Ematologia 3 J, Policlinico Gemelli, Largo Gemelli 8, Roma, 00168 Italy

**Keywords:** Pomalidomide, Hypothyroidism, Multiple myeloma

## Abstract

Pomalidomide, an analogue of lenalidomide and thalidomide, has shown significant activity in treating relapsed refractory multiple myeloma (MM). Due to its oral formulation and favorable safety profile, pomalidomide is generally well tolerated, even among elderly and frail patients. We present a case of severe hypothyroidism induced by pomalidomide in a 71-year-old woman with relapsing/refractory MM. After initiating levothyroxine therapy, her symptoms quickly resolved. We then attempted to resume pomalidomide treatment; the rechallenge proved safe concerning thyroid function. In conclusion, we recommend testing thyroid function in patients prior to and then every 2–3 months during pomalidomide treatment. If drug-induced hypothyroidism occurs, once the patient reaches a state of euthyroidism, a rechallenge can be considered with a good safety profile.

## Introduction

Multiple Myeloma (MM) is characterized by frequent relapse, requiring most patients to undergo several lines of therapy [1]. Recently, new drugs with diverse mechanisms have significantly improved outcomes, even in relapsed cases.

MM primarily affects older adults, with a median diagnosis age of 69. Aggressive treatments are often less effective and poorly tolerated in elderly or frail patients, resulting in higher morbidity and mortality [2].

Selecting the most effective and safe treatment for each patient is a clinical challenge. This decision depends on comorbidities, disease characteristics, and prior therapy exposure and response in cases of relapse.

Pomalidomide, an analogue of lenalidomide and thalidomide, is effective in relapsed and refractory MM [3, 4]. Its oral formulation and favorable safety profile make it generally well tolerated. The response rate for pomalidomide with dexamethasone (Pd) in patients refractory to lenalidomide and bortezomib is about 30%, and increases significantly when combined with other drugs [5, 6].

Pomalidomide-based triplet regimens are important options for relapsed patients who are lenalidomide-refractory, especially when combined with proteasome inhibitors such as bortezomib and anti-CD38 monoclonal antibodies [7–10].

The combination of isatuximab, pomalidomide, and dexamethasone (Isa-Pd) improves progression-free survival (PFS) compared to Pd in patients with relapsed/refractory MM, with median PFS of 11.5 months versus 6.5 months [7, 8]. A sub-analysis of the ICARIA-MM study (Isatuximab-pomalidomide-dexamethasone versus pomalidomide-dexamethasone in patients with relapsed and refractory MM) found that Isa-Pd’s clinical response and safety in frail patients were consistent with the overall population, supporting its feasibility for this group [11].

## Case description

We present the case of a 71-year-old Caucasian woman with relapsed/refractory MM. She was diagnosed with IgG kappa MM at age 63 in 2017, with hypercalcemia, multiple bone lesions, osteoporosis, and dyslipidemia.

She initially received two cycles of Bortezomib-Thalidomide-Dexamethasone (VTD) with the goal of autologous bone marrow transplantation. This plan was postponed due to multiple respiratory infections and hospitalization for sialoadenitis.

She then started second-line therapy with Bortezomib-Melphalan-Prednisone (VMP), achieving serological partial remission and improvement in lytic lesions.

In 2020, she experienced a serological relapse, with a gradual increase in serum monoclonal component and free light chain (FLC) kappa. Third-line treatment with Elotuzumab, Lenalidomide, and Dexamethasone was initiated. During this period, she developed peripheral neurological toxicity, diabetes managed with metformin and insulin, and bilateral lower limb deep vein thrombosis, requiring long-term apixaban therapy.

In 2021, upon her diabetologist’s recommendation, she underwent a thyroid echo-doppler, which showed no pathological alterations.

By February 2023, she experienced another relapse, with neutropenia caused by over 50% plasma cell infiltration and further increases in serum monoclonal component and FLC kappa. Blood and bone marrow tests, including FISH, showed t(11;14) and 1q21 gain. Thyroid function was normal: TSH was 2 µUI/ml (reference range: 0.35–3.80) and FT3 was 3.5 pg/ml (reference range: 2.4–4.2).

She began fourth-line therapy with Isatuximab, Pomalidomide, and Dexamethasone (IPD). Her home medications included omeprazole, trimethoprim/sulfamethoxazole, acyclovir, pregabalin, apixaban, and metformin. After the first Pomalidomide cycle, she developed grade 4 febrile neutropenia, leading to a dose reduction from 4 mg to 3 mg daily. The steroid dose was also reduced due to intolerance and recurrent respiratory infections. In March 2024, she was admitted to the intensive care unit for pneumonia with lung failure. Despite therapy delays due to infections, she achieved a partial response with reduced serum disease markers.

In December 2024, pre-therapy evaluation revealed moderate edema in her lower limbs and face, without other significant symptoms. Diuretics were prescribed, and chemotherapy was administered.

In January 2025, her symptoms worsened, including drowsiness for over half the day, impaired speech and mobility, mood changes, constipation, and increased edema.

The differential diagnosis included MM progression with extramedullary or cerebral involvement, cerebral vein thrombosis given her history of leg thrombosis, major depression following her husband’s recent death, and hypothyroidism.

Thyroid tests showed TSH at 189 microUI/ml (reference range: 0.35–3.80), FT3 at 0.5 pg/ml (reference range: 2.4–4.2), and FT4 at 1.2 pg/ml (reference range: 8.5–16.5). MM serum markers were slightly elevated, with IgG at 856 mg/dl compared to 670 mg/dl in September 2024. An urgent endocrinology evaluation determined hospitalization was necessary. She started levothyroxine replacement at 0.6 mcg/kg, resulting in immediate clinical improvement. The dose was increased to 1.5 mcg/kg over the following days.

During hospitalization, a thyroid ultrasound showed normal morphology and volume, but a diffusely irregular echostructure with a paravascular appearance and fibrous strands, possibly indicating thyroiditis. Anti-thyroglobulin (TG) and thyroperoxidase (TPO) antibodies were within normal limits.

A thyroid biopsy was not performed. Therefore, histological data are not available.

The endocrinologist concluded that pomalidomide was the most likely cause of the patient’s hypothyroidism based on several observations. Thyroid function tests, including TSH and FT3, were within normal limits before the patient began the IPD regimen. At the time of hypothyroidism diagnosis, hormone levels suggested chronic toxicity. As noted in the discussion, thyroid toxicity has been reported with other medications in the same class as pomalidomide, supporting this association. The patient’s other medications are not known to affect thyroid function.

Autoimmune thyroiditis was considered unlikely because blood tests for anti-TG and anti-TPO antibodies, which are typically elevated in Hashimoto’s disease, were negative.

There were also no clinical signs or symptoms of an infectious process affecting the thyroid. Based on these findings, the endocrinologist recommended temporarily discontinuing chemotherapy until the patient’s thyroid function normalized.

By March 2025, thyroid blood tests showed TSH at 0.70 microUI/ml (reference range: 0.35–3.80), FT3 at 4.0 pg/ml (reference range: 2.4–4.2), and FT4 at 15 pg/ml (reference range: 8.5–16.5). MM markers remained stable, with IgG at 687 mg/dl, FLC kappa at 420 mg/L (reference range: 3.3–19.4), and a FLC k/λ ratio of 205. As MM was stable following therapy interruption, the endocrinologist recommended resuming the same chemotherapy regimen three months after the previous administration and continuing levothyroxine.

The patient resumed pomalidomide based on several factors. She had an indolent disease that responded well to pomalidomide, had experienced multiple infections requiring hospitalization during more aggressive treatments, and achieved rapid restoration of euthyroidism with complete symptom resolution after starting levothyroxine replacement therapy.

In April 2025, thyroid blood tests showed: TSH at 0.21 microUI/ml (reference range: 0.35–3.80), FT3 at 3.7 pg/ml (reference range: 2.4–4.2), and FT4 at 16 pg/ml (reference range: 8.5–16.5).

By May 2025, blood tests showed MM progression, with FLC kappa increasing to 1126 mg/L (reference range: 3.3–19.4) and a FLC k/λ ratio of 433. Thyroid function remained normal, and there were no clinical signs of MM. She is now being evaluated for a fifth line of therapy, with bispecific antibodies or CAR-T cell therapy under consideration.

## Discussion

Pomalidomide, a third-generation immunomodulatory drug (IMiD), acts by binding to the intracellular protein Cereblon. This interaction promotes the ubiquitination and degradation of key transcription factors, including Aiolos, Ikaros, Ck-1, and GSPT1, resulting in antiangiogenic and anti-proliferative effects. Pomalidomide also enhances the anti-tumor immune response by costimulating T cells and increasing the activity and proliferation of natural killer (NK) and natural killer T (NKT) cells. These mechanisms are shared with thalidomide and lenalidomide. However, unlike thalidomide, pomalidomide reduces levels of regulatory T-cells (Tregs) and Th17 cells [12].

Several antineoplastic drugs, particularly those with immunomodulating effects, are known to cause hypothyroidism. In hematology, the most common agents include interferons, tyrosine kinase inhibitors (such as imatinib and dasatinib), thalidomide, and lenalidomide, each with distinct mechanisms of action [13]. Up to 14% of patients treated with thalidomide for MM develop subclinical or clinical hypothyroidism [14, 15]. A retrospective study found that up to 6% of patients receiving lenalidomide (88% with MM) without prior thyroid dysfunction were diagnosed with hypothyroidism, and some with hyperthyroidism [16]. Among patients with pre-existing thyroid dysfunction, lenalidomide therapy was associated with a higher incidence of abnormal thyroid function tests (17%) compared to those without prior dysfunction (6%) (*P* = 0.0001) [16].

Proposed mechanisms for IMiD-induced hypothyroidism include T-cell stimulation, antiangiogenic activity, and cytokine release, which may lead to ischemic or autoimmune thyroiditis [14–17]. For lenalidomide, hypothyroidism typically develops between 1 and 6 months after initial exposure, with a median onset of 5 months. This timeframe may differ for other immunomodulating drugs [13].

Reported side effects of pomalidomide include neutropenia, anemia, thrombocytopenia, infections, fatigue, nausea, fever, diarrhea, thromboembolic events, skin toxicities, hyperglycemia, hypokalemia, and peripheral neuropathies. Notably, hypothyroidism has not been identified as a side effect in major clinical studies [3–11].

A few case reports suggest a possible correlation between pomalidomide and hypothyroidism. For example, a 51-year-old woman with relapsed/refractory MM developed clinical hypothyroidism after four cycles of pomalidomide [18]. An 83-year-old woman with the same condition experienced symptoms after four weeks of treatment [19], and a 70-year-old man presented with associated hyponatremia [20] (Table 1).

**Table 1:** Comparison between reported cases of pomalidomide-induced hypothyroidism

Pomalidomide-induced hypothyroidism	Reference [18]	Reference [19]	Reference [20]	Present case
Age	51	83	70	71
Male/female	F	F	M	F
MM Type	IgG kappa	IgG kappa	IgG kappa	IgG kappa
Line of MM therapy	3rd	4th	2nd	4th
Time of onset	16 weeks	4 weeks	32 weeks	104 weeks
MM status	NR	NR	NR	PR
TSH level	175 mUI/L (range NR)	44.7 mUI/L (range 0.35–4.94)	88.6 mUI/L (range 0.38–4.70)	189 mUI/L (range 0.35–3.80)
Hyponatremia	No	No	Yes	No
Anti-TPO antibodies	NR	Yes	Yes	No
Response to levothyroxine	Yes	Yes	Yes	Yes
Pomalidomide treatment	Discontinued	Discontinuation not reported	Discontinuation not reported	Resumed after discontinuation

*NR* not reported, *PR* partial remission

To assess the potential causal relationship between hypothyroidism and pomalidomide, we applied the Naranjo Adverse Drug Reaction Probability Scale, a standard tool in pharmacovigilance. The result was 7 points, indicating a probable association. This score reflects that similar reactions have been reported in case studies, the adverse event occurred after drug exposure and resolved quickly after discontinuation, no alternative causes were identified, and the diagnosis was confirmed by blood tests and ultrasonography [21]. 

We searched for real-world and pharmacovigilance data on the association between pomalidomide and hypothyroidism but found no relevant results, despite hypothyroidism being a known adverse event of other IMiDs. We believe that hypothyroidism related to pomalidomide is significantly underdiagnosed and underreported.

The cases discussed differ in patient age, MM status, and biochemical parameters, yet all show similar symptoms and respond promptly to levothyroxine therapy. In our case, notable features include a severe clinical presentation, late symptom onset after two years of therapy, which has not been previously reported, and MM progression after a required three-month treatment interruption.

Regarding the late onset of hypothyroidism after starting treatment, we believe the delay between hypothyroidism diagnosis and pomalidomide initiation is likely due to cumulative effects and delayed diagnosis. The patient had few symptoms and did not receive regular endocrine monitoring.

The absence of anti-thyroid antibodies, unlike in other reported cases, suggests possible dysregulation of cell-mediated immunity in addition to other IMiD-induced mechanisms.

Previous cases did not specify how MM treatment was managed during this period. In one report, treatment with pomalidomide was resumed after hypothyroidism was addressed. In our case, we also resumed pomalidomide after achieving euthyroidism, based on the indolent, partially remitted MM and the patient’s overall tolerability aside from hypothyroidism. Ttreatment efficacy declined, but no new thyroid toxicity occurred.

In conclusion, we recommend assessing thyroid function before initiating pomalidomide therapy and every three months during treatment, as previously suggested [18] (Table 2). Clinicians should monitor for symptoms to enable early diagnosis and prevention of severe hypothyroidism. Prompt levothyroxine treatment can prevent severe hypothyroidism and allow uninterrupted pomalidomide therapy. This approach is especially important for frail and elderly patients, who are at higher risk of complications. It also preserves pomalidomide as a valuable and well-tolerated treatment option, as shown in our case. After stabilizing thyroid function and achieving euthyroidism, we resumed pomalidomide without further thyroid dysfunction. Therefore, reinitiating pomalidomide may be feasible if it remains the best option for the patient’s MM.

**Table 2:** Recommendations for MM patients receiving IMiDs

1. Assess thyroid function before initiating treatment with IMiDs.
2. Monitor thyroid function every three months during IMiD treatment.
3. If thyroid function tests are abnormal, perform echography and measure anti-thyroid antibodies.
4. A diagnosis of hypothyroidism, whether before or during IMiD treatment, does not require discontinuing multiple myeloma therapy, as hormonal supplementation is an effective option.

## Data Availability

No datasets were generated or analysed during the current study.
